# In COVID-19 Patients Supported with Extracorporeal Membrane Oxygenation, Intensive Care Unit Mortality Is Associated with the Blood Transfusion Rate

**DOI:** 10.3390/jcm13237381

**Published:** 2024-12-04

**Authors:** Maged Makhoul, Eldad J. Dann, Tatiana Mashiach, Oleg Pikovsky, Roberto Lorusso, Jamela Eisa, Halil I. Bulut, Ori Galante, Eduard Ilgiyaev, Gil Bolotin, Naomi Rahimi-Levene

**Affiliations:** 1 Department of Cardiac Surgery, Rambam Health Care Campus, Haifa 3109601, Israel; magedmakhoul@gmail.com (M.M.); g_bolotin@rambam.health.gov.il (G.B.); 2 The Ruth and Bruce Rappaport Faculty of Medicine, Technion, Israel Institute of Technology, Haifa 3109601, Israel; 3 Blood Bank and Apheresis Unit, Rambam Health Care Campus, Haifa 3109601, Israel; tania.mashiah@gmail.com (T.M.); j_eisa@rambam.health.gov.il (J.E.); 4 Transfusion Medicine and Apheresis Institute, Soroka University Medical Center, Faculty of Health Sciences, Ben Gurion University of the Negev, Beer-Sheva 84101, Israel; olegpi@clalit.org.il; 5 Department of Cardio-Thoracic Surgery, Heart and Vascular Centre, Maastricht University Medical Centre, 6229 HX Maastricht, The Netherlands; roberto.lorusso@mumc.nl; 6 Cerrahpasa School of Medicine, Istanbul University Cerrahpasa, Istanbul 34098, Turkey; halilibrahim.bulut@ogr.iuc.edu.tr; 7 Medical Intensive Care Unit, Soroka University Medical Center, Faculty of Health Sciences, Ben Gurion University of the Negev, Beer-Sheva 84105, Israel; origa@clalit.org.il; 8 Intensive Care Department, Shamir Medical Center, Zerifin 7033001, Israel; 9 Adelson School of Medicine, Ariel University, Ariel 4070000, Israel; nrlevene@shamir.gov.il; 10 Blood Bank, Shamir Medical Center, Zerifin 707300, Israel

**Keywords:** acute respiratory distress syndrome, COVID-19, extracorporeal membrane oxygenation, packed red blood cell transfusion

## Abstract

**Background**: The COVID-19 pandemic markedly increased the number of patients with infection-related acute respiratory distress syndrome who required extracorporeal membrane oxygenation (ECMO) and multiple blood transfusions. This study aimed to assess a potential correlation between the daily rate of transfused blood products and the intensive care unit (ICU) outcome of ECMO-supported COVID-19 patients. **Methods**: Data were retrieved from the electronic databases of three Israeli tertiary care centers. All COVID-19 patients treated with ECMO for >3 days in these centers between July 2020 and November 2021 were included in the analysis. **Results**: The study incorporated 106 patients [median age 49 (17–73) years]. The median numbers of ECMO days and daily transfused packed red blood cell (PRBC) units were 20.5 (4–240) and 0.61 (0–2.82), respectively. In multivariate analysis, age ≥50 years was an independent factor for ICU mortality [odds ratio (OR) 4.47). In ECMO-supported patients for <38 days, transfusion of ≥0.85 units/day was associated with higher ICU mortality compared to that observed in patients transfused with <0.85 PRBC units/day (OR = 5.43; *p* < 0.004). Transfusion of ≥0.5 units/day combined with ECMO support of ≥38 days (OR = 17.9; *p* < 0.001) conferred the highest mortality risk. **Conclusions**: Three-quarters of patients <50 years old and half of patients ≥50 years were successfully discharged from ICU. Higher daily transfusion rates were associated with significantly increased ICU mortality, irrespective of ECMO duration. Reduced blood transfusion may improve the survival of these patients. This approach could also contribute to the measures taken to address the challenges of blood shortages occurring during pandemics and other global or national emergencies.

## 1. Introduction

Acute respiratory distress syndrome (ARDS) secondary to COVID-19 [1] is a severe and life-threatening complication [2] with limited treatment options. The use of extracorporeal membrane oxygenation (ECMO) in patients with COVID-19-associated ARDS is recommended by the guidelines of the World Health Organization and the Extracorporeal Life Support Organization (ELSO) [3,4]. Multiple national and international studies that analyzed the outcomes of such patients during various waves of the pandemic, with the intent of identifying patients for whom ECMO support would be most beneficial, suggested consideration of its use in COVID-19 patients with refractory respiratory failure [5,6,7,8,9]. The vast majority of these patients are severely hypoxemic and need adequate levels of hemoglobin to maintain the oxygen capacity of blood as well as oxygen delivery. Due to bleeding complications and hemolysis, transfusions of packed red blood cells (PRBCs) and other blood products are often required during ECMO support [10,11,12], although this is known to be frequently associated with an unfavorable patient prognosis [13]. In the pre-COVID-19 era, the standard of care was to keep patient hemoglobin levels within the range of 8–10 g/dL. The international TRAIN-ECMO survey, evaluating transfusion practice based on the data provided by 447 ECMO centers worldwide, found the preferential use of a lower hemoglobin threshold [(8.4 mg/dL (95% CI: 7.7–8.9) versus 8.9 mg/dL (95% CI: 8.2–9.7)] at high-volume centers [14]. A retrospective comparison between restrictive and liberal transfusion strategies employed in ECMO-supported patients found a similar intensive care unit (ICU) outcome when a hemoglobin transfusion threshold of 8.5 g/dL was used [15]. The guidelines by the European Society of Intensive Care Medicine recommended using restrictive (7 g/dL) and not liberal (9 g/dL) hemoglobin blood transfusion thresholds for patients with or without ARDS treated in ICUs [16]. Yet, due to the lack of sufficient research findings, these guidelines were not applicable to critically ill adults undergoing veno-venous (VV) or veno-arterial (VA) ECMO. In a retrospective matched cohort study comparing outcomes of adult ARDS patients treated with either the restrictive or liberal transfusion approach, including 99 patients treated with VV-ECMO, the transfusion at a hemoglobin threshold of 8 g/dL was not found to be associated with higher 28-day mortality compared with that observed in patients transfused at the threshold of 10 g/dL [17].

During the COVID pandemic, while the updated ELSO guidelines [18] recommended no changes in the transfusion strategy for ECMO-supported COVID-19 patients compared to that applied for other indications, due to the shortage of blood products, the restrictive transfusion approach started to be commonly applied, showing non-inferior outcomes relative to those associated with the use of the liberal approach in retrospective analyses [19,20]. The study conducted by the research group from Belgium, using the restrictive transfusion strategy with the hemoglobin cutoff <7 g/dL, demonstrated that even with this approach, about 80% of patients on VV-ECMO required the transfusion of a median of 0.5 PRBC units/day [21].

Another issue that has an impact on COVID-19 patient outcomes is the duration of the ECMO support. A multi-center UK study, analyzing both blood component use and VV-ECMO duration in COVID-19 patients compared to non-COVID-19 patients, reported significantly longer ECMO support and lower PRBC requirements among patients from the former group [19]. Notably, a restrictive transfusion protocol was applied in both groups.

While a number of studies evaluated the hemoglobin cutoff for blood transfusion in this vulnerable patient population [19,20,22], there is a paucity of data regarding an association between the amount of transfused blood and ICU patient outcomes during the COVID-19 pandemic.

Hence, this study aimed to evaluate potential associations between the total and daily PRBC transfusion rate and ICU mortality in ECMO-supported COVID-19 patients, as these findings could be beneficial to a wider range of patient populations suffering from ARDS caused by other severe respiratory infections.

## 2. Materials and Methods

All COVID-19 patients requiring ECMO support were recorded in the National Israeli Research Electronic Data Capture (REDCap^®^) Registry. The conduction of this retrospective cohort study was approved by the Institutional Review Board (IRB) of each of the three participating Israeli tertiary care centers (Rambam Health Care Campus, Soroka University Medical Center, and Shamir Medical Center). Following IRB approval, data on all COVID-19 patients treated with ECMO for >3 days in these centers between July 2020 and November 2021 were retrieved from institutional electronic databases and included in the analysis. The collected data incorporated patient demographics, ECMO parameters, the total and daily amounts of transfused PRBC, fresh frozen plasma (FFP), and cryoprecipitate units, as well as ICU mortality during ECMO support and post-weaning. The indications for ECMO support applied in this study were based on the ELSO guidelines [23] and were previously described in detail by Makhoul et al. [24].

The primary endpoint was ICU mortality. The secondary endpoints were associations between the daily rate of transfused PRBC units, patient age, ECMO duration, and ICU mortality.

### Statistical Analysis

The exposure variables of this study were patient age, ECMO support duration, and the total amounts and daily rates of transfused PRBCs and FFP.

Baseline characteristics were analyzed with standard descriptive statistics. Associations between the patient age, ECMO support duration, transfused blood components, and ICU mortality were assessed using the Mann–Whitney non-parametric test. The area under the receiver operating characteristic (ROC) curve and Youden’s J statistics were applied to define cutoff points for the amount of transfused blood, ECMO support duration, the daily transfusion rate, and its association with ICU mortality. The bivariate logistic regression analysis was used to calculate odds ratios (OR) with 95% confidence intervals (CI) and *p*-values for factors associated with ICU mortality. The multivariate stepwise logistic regression analysis was performed to assess the weight of the parameter of interest (the daily transfusion rate) compared to the weight of other variables associated with ICU mortality. The area under the ROC curve was used to measure model discrimination. Two-tailed *p*-values ≤0.05 were considered statistically significant. Statistical analyses were performed with IBM SPSS Statistics for Windows (Version 28.0) IBM Corp (Armonk, NY, USA) (2021).

## 3. Results

Overall, 106 consecutive patients who were on ECMO due to severe COVID-19-related ARDS were included in the study. Four patients who were on ECMO support for ≤3 days were excluded from the analysis because of the short exposure to ECMO. Of the 102 analyzed patients [median age 49 years (17–73); 25 and 77 treated in 2020 and 2021, respectively], 76.5% (*n* = 78) were males (Table 1). During the study period, the liberal blood transfusion strategy, with the hemoglobin cutoff value of 10 g/dL, was applied to VV-ECMO-supported patients in the participating centers.

The median number of ECMO days was 20.5 (4–240) and the median transfusion rate was 0.61 (0–2.82) PRBC units/day (Table 2).

The vast majority of patients (89%; *n* = 91) suffered from COVID-19-associated respiratory failure requiring lung support only and were treated with VV-ECMO. VA-ECMO was applied to the patients who had both respiratory and cardiac failure (11%; *n* = 11). The total ICU mortality rate was 39%, with rates of 48% and 36% in 2020 and 2021, respectively (OR = 0.62; 95% CI: 0.2–1.5; *p* >0.05), and on-ECMO mortality of 26.5%. Successful ECMO weaning was achieved in 73.6% (*n* = 75) of patients; however, 12.5% (*n* = 13) of them died in the ICU post-weaning. On-ECMO mortality and ICU mortality rates for patients on VV-ECMO and VA-ECMO were 23.1% and 54.5% versus 36% and 64%, respectively. The median age of patients who survived in ICU was 45.5 years (17–73) versus 54.5 years (20–69) for those who died there (*p* = 0.001) (Table 2).

In a bivariate analysis of patient outcome, the stratification by the age cutoff of 50 years demonstrated significantly greater ICU mortality for those ≥50 (56%) compared to individuals <50 years old (23%; Table 3). There were no significant differences between the two age groups in terms of ECMO support duration, the daily rate of PRBC transfusion, and the total amount of transfused PRBC, FFP, or cryoprecipitate units (Table 1).

Study participants were divided into the following groups according to the rate of transfused PRBC units/day during ECMO support: ≤0.5; >0.5 and <0.85; ≥0.85. The OR for ICU mortality was significantly higher in patients transfused with ≥0.85 PRBC units/day relative to those transfused with ≤0.5 units/day (OR = 4.96; *p* < 0.002) (Table 3). The total amount of transfused PRBC units/patient was found to be significantly lower in ICU survivors compared to non-survivors, with median amounts being 8.0 (0–98) and 29.5 (2–197) units, respectively (*p* = 0.001) (Table 2). Likewise, the FFP transfusion significantly differed between the groups (*p* = 0.001). In the ROC analysis for the total amount of transfused PRBC units/patient, the transfusion of ≥16 units were found to be associated with higher ICU mortality, with the area under the curve (AUC) of 0.74 (95% CI 0.64–0.84). For the transfusion rate of >0.85 units/day, the area under the ROC curve was 0.698 (95% CI 0.553–0.884).

There was a significant difference in the patient ICU outcome based on the duration of ECMO support. Patients who survived in the ICU (*n* = 62) were ECMO-supported for a median of 13.5 days, while the corresponding number for patients who died during the ICU stay (*n* = 40) was 38.5 days (*p* = 0.005) (Table 2). In the ROC analysis, the ECMO duration of ≥38 days was found to be associated with higher mortality, with an AUC of 0.66 (95% CI 0.55–0.77). The bivariate analysis demonstrated significantly higher ICU mortality among the patients supported with ECMO for ≥38 days (OR = 4.6; 95% CI 1.9–11.2; *p* < 0.001) in both age groups (<50 years and ≥50 years) (Table 3).

Among the 45 patients, who were transfused with <0.85 PRBC units/day and ECMO-supported for <38 days, the ICU mortality rate was 16% (OR = 1.00). In the group of 24 patients, transfused with ≥0.85 PRBC units/day and being on ECMO for <38 days, the ICU mortality rate amounted to 50% (*p* < 0.004) (Table 3). Patients who were supported on ECMO for ≥38 days and were transfused with >0.5 PRBC units/day (*n* = 21) had an ICU mortality rate of 76.2% (OR = 17.37; 95% CI 4.79–62.96; *p* < 0.000).

**Figure 1:**
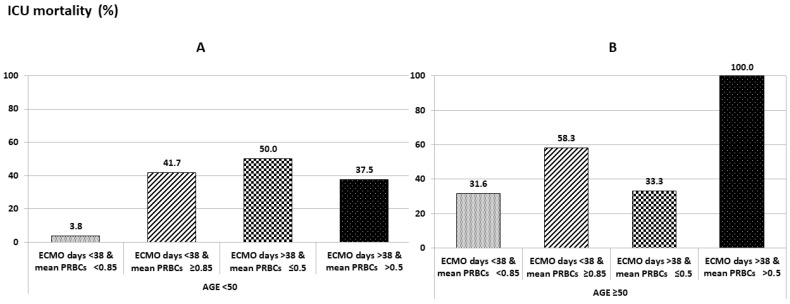
ICU mortality according to patient age, the rate of transfused PRBC units/patient/day, and ECMO support duration. (**A**) ICU mortality in patients <50 years of age according to the mean rate of transfused PRBC units/patient/day and ECMO support duration. (**B**) ICU mortality in patients ≥50 years of age according to the mean rate of transfused PRBC units/patient/day and ECMO support duration.

In the multivariate analysis, ECMO support of ≥38 days and transfusion of ≥0.5 PRBC units/patient/day as well as patient age ≥50 years all emerged as adverse prognostic factors for increased ICU mortality. Notably, age ≥50 years was identified as a significant independent factor (*p* = 0.0003) (Table 4, Figure 1).

The ECMO support duration was not found to be an independent adverse variable; however, high ICU mortality, associated with both increased PRBC transfusion rate and long ECMO support, was observed in both age groups. Patients who were transfused with ≥0.5 PRBC units/day and were on ECMO for ≥38 days had an OR for ICU mortality of 17.9 (95% CI 4.57–70.26) compared to patients who required <0.85 units/day of PRBC and <38 days of ECMO support (*p* < 0.001) (Table 4). The ROC of this multivariate model was 0.809 (0.7–0.9).

In the current study, 75/102 (73.6%) patients were successfully weaned from ECMO, 13 (17%) of whom died post-weaning. The most frequent on-ECMO and in-ICU cause of death after ECMO weaning was sepsis, amounting to 64% and 46%, respectively (Table 5). The sepsis-related causes of death in patients on ECMO included ventilator-associated pneumonia (VAP) (*n* = 5), urinary tract infections (UTI) (*n* = 1), cellulitis (*n* = 1), candidemia (*n* = 2), and bacteremia (*n* = 4). Such data were unavailable for three patients. The sepsis-related causes of death in patients who succumbed in the ICU post-weaning were VAP (*n* = 3), candidemia (*n* = 2), and bacteremia (*n* = 1).

Other on-ECMO causes of death were cardiac, bleeding, neurological, and mechanical complications. Other causes of death after ECMO weaning were brain death, multi-organ failure (MOF), and respiratory failure. Sixty-two (60.7%) patients were successfully discharged from the ICU.

## 4. Discussion

ECMO support has been recommended by the ELSO for patients with refractory respiratory failure secondary to the COVID-19 infection [18]. However, high on-ECMO mortality calls for the investigation of its causes and risk factors [20,25]. The major issues to be explored in this context include a potential association between the amount of blood received by patients during ECMO support and ECMO ICU outcomes. This is also important, as the blood supply became limited during the pandemic [12,26].

In the pre-COVID-19 era, ECMO support was shown to improve survival in patients with ARDS secondary to pulmonary infections [27]. In a previous study conducted by our group evaluating outcomes of 197 COVID-19 patients receiving VV-ECMO support during the first year of the pandemic, the overall in-hospital mortality rate was 54% [24]. In the current study, including eight patients from the earlier analysis, the overall ICU mortality of 39% was documented, which is in line with data from large meta-analyses and other multi-center studies [28,29,30].

In our study, the ICU mortality was more than two-fold higher in patients older relative to those younger than 50 years. The age ≥50 years was found to be an independent risk factor for increased mortality both in bivariate and multivariate analyses. This finding was solid given that neither the duration of ECMO support nor the amount of transfused blood differed between the two age groups (<50 and ≥50 years). Similarly, Lorusso et al. observed a significant association between older age and increased in-hospital mortality in a multicenter study, including 1215 COVID-19 patients who were on ECMO support during the first year of the pandemic [25]. In that study, the mortality rate in patients aged 60–69 years was significantly higher than in patients younger than 60 years. Of note, this age cutoff was a decade older than that applied in our study (Table 3). Likewise, a large study from France including 429 patients (median age of 54 years), supported with VV-ECMO, identified increased age as an independent risk factor for mortality in this patient population [31].

Bleeding, hemolysis, and other clinical scenarios frequently occurring in ECMO patients necessitate blood transfusion, which per se is known to be associated with immunological and non-immunological adverse effects. There are no clear evidence-based recommendations or consensus expert opinions regarding the management of blood transfusion and its effect on the survival of ECMO-supported COVID-19 patients [20]. In 2021, Hughes et al. published a meta-analysis of 54 studies assessing the PRBC transfusion practice in adult ECMO patients in the pre-COVID-19 era [32]. In that cohort of 1349 patients, data on VV-ECMO support were available for 665 patients. The mean total amount of transfused PRBC units/patient on VV-ECMO support was 19.3 (95% CI 10.4–28.1) or 1.23 (95% CI 0.89–1.57) units/day. This meta-analysis demonstrated the heterogeneity in the PRBC transfusion practice, and suggested an association between increased PRBC transfusion rates and an inferior survival, while the direction and strength of such an association remained uncertain. In 2016, Mazzeffi et al. published the transfusion data on 132 patients, 64 of whom were treated on VV-ECMO [13]. In that study, a median transfusion rate of 15 (9–25) PRBC units was reported, and multivariate logistic regression analysis identified increased transfusion as the only significant factor for in-hospital mortality, with an OR of 1.03 (95% CI 1–1.06, *p* = 0.04).

During the COVID-19 pandemic, several studies assessed mortality risk factors in patients with ARDS treated with ECMO. In a retrospective study including 100 patients suffering from ARDS secondary to COVID-19, univariate analysis showed a significant association of higher 60-day mortality with several parameters, including the number of transfused blood units [33]. The data from both pre-COVID-19 and the pandemic periods are in line with the findings of the present study, where a bivariate analysis identified the total amount of transfused PRBC units/patient of ≥16 as a highly significant risk factor for increased ICU mortality (*p* < 0.001).

The median number of ECMO support days in the current study was 20.5, while in one-third of the patients, the ECMO duration was ≥38 days, which was associated with significantly higher ICU mortality in the latter group. However, neither the duration of ECMO nor the PRBC daily transfusion rate alone emerged as independent risk factors in a multivariate analysis. On the other hand, the transfusion of ≥0.5 PRBC units/patient/day in combination with ≥38 days on ECMO was found to be associated with a significantly higher ICU mortality rate relative to that observed in patients who required <0.85 PRBC units/day and <38 days of ECMO support. Remarkably, patients younger than 50 years who were on ECMO for <38 days demonstrated a dramatic increase in ICU mortality associated with elevated daily transfusion rates (≥0.85 PRBC units). Hence, increased ICU mortality was associated with exposure to a higher daily transfusion rate or a higher total amount of transfused PRBC units/patient. Given these findings, major efforts need to be undertaken to reduce the exposure of such patient populations to PRBC transfusion.

Over the last few years, the application of restrictive transfusion protocols has become the recommended standard of care for ICU patients [34], while the implementation of this recommendation in the setting of ECMO patients remains quite limited. The pre-COVID-19 observational study by Ng et al., analyzing the ICU outcome of VV-ECMO-supported patients for whom the cutoff value between the liberal (*n* = 317) and restrictive (*n* = 65) transfusion was set at 8.5 g/dL, demonstrated that the restrictive strategy did not yield a worse outcome [15].

An expert opinion consensus report advocating for the use of restrictive transfusion for ECMO-supported COVID-19 patients was published by Canadian extracorporeal life support experts [35]. The cohort study from the University of Minnesota Medical Center that reviewed the outcome of 507 patients, including 70 supported with VV-ECMO, found non-inferiority in survival rates between ECMO patients treated with a restrictive (hemoglobin threshold 7 g/dL; *n* = 34) or liberal (hemoglobin threshold 10 g/dL; *n* = 36) transfusion protocol [20]. However, the relatively small size of that VV-ECMO subgroup precluded definitive conclusions. Importantly, a large prospective multicenter observational study evaluating the transfusion practice in COVID-19 VV-ECMO patients (PROTECMO) demonstrated a highly significant benefit of PRBC transfusion for 28-day survival only in patients with a hemoglobin level <7 g/dL [22].

Along with the application of the restrictive transfusion approach, measures need to be taken to minimize the impact of iatrogenic blood loss due to frequent daily blood sampling for laboratory testing, which eventually necessitates increased blood transfusion in the ICU patient population. The recently published STRATUS randomized trial suggested that the utilization of small-volume blood collection tubes might reduce the number of PRBC transfusions in this clinical setting [36]. Importantly, all these measures could also be beneficial in terms of effective blood supply management in settings with both resource limitations and high demand for blood products [21,26].

In the present study, 73.6% of all patients were successfully weaned from ECMO and 60.7% of all patients were subsequently discharged from the ICU. In the current cohort, sepsis was found to be the most common cause of death both on ECMO and in the ICU after weaning. In the previously reported systematic review of studies analyzing patients on VV-ECMO support in the pre-COVID-19 era, MOF and sepsis were identified as the most common causes of death on ECMO and during the hospital stay post-ECMO weaning [37,38]. During the pandemic, a large prospective, multicenter study on ECMO support used for patients with COVID-19 demonstrated an in-hospital mortality of 50%. In that study, the most common in-hospital causes of death were MOF (36%), respiratory failure (27%), and sepsis (12%) [25]. The predominance of sepsis as the cause of death in the current study is explained by the fact that COVID-19 patients continue to be fragile and prone to sepsis even after partial lung recovery, respiratory stabilization, and ECMO weaning. Another possibility is that steroids routinely administered to such patients make them more vulnerable to hospital-acquired infections. This is in line with a report on residual abnormal respiratory function in patients recovering from COVID-19 who have not required ECMO support [39].

This study has several inherent limitations that could be attributed to its retrospective design, such as selection bias and missing information. However, this study included all consecutive COVID-19 patients, treated with ECMO in the participating centers for >3 days, which excludes the potential of selection bias. Since all the data were collected from the National ECMO database or directly from blood banks, the unavailability of certain clinical information regarding each patient included in the study is a possible limitation of this analysis. For instance, missing data regarding patient comorbidities or technical problems faced during ECMO support could contribute to patient variability in rates of transfused PRBC units. Additionally, frequently, the liberal transfusion strategy (hemoglobin cutoff value 10 g/dL) has been applied, which is not the current common practice.

## 5. Conclusions

In the current study, three-quarters of ECMO-supported COVID-19 patients younger than 50 years and about half of older patients survived the ICU hospitalization. The combination of longer ECMO support and a higher daily rate of transfused PRBC units was found to be significantly associated with increased ICU mortality in both age groups. Given these findings, the decrease in blood transfusion might be beneficial. This could be achieved through using the restrictive transfusion strategy and minimizing the iatrogenic blood loss. Randomized controlled trials are warranted to obtain a higher level of evidence that could potentially result in the efficient management of ECMO patients in future pandemics.

## Figures and Tables

**Table 1:** ECMO and blood transfusion parameters analyzed according to patient’s age, using 50 years as a cutoff.

Parameters		Patients, Total; *n =* 102 (%)	Age < 50 Years; *n* = 52 (%)	Age ≥ 50 Years; *n* = 50 (%)	*p* Value
Gender	Male	78 (76)	38 (73)	40 (80)	0.487
	Female	24 (24)	14 (27)	10 (20)	
Type of ECMO	VV	91 (89.3)	46 (88)	45 (90)	0.09
	VA	11 (10.7)	6 (12)	5 (10)	
ECMO days	<5	2 (1.9)	1 (2)	1 (2)	0.252
	5–7	14 (13.7)	9 (17)	5 (10)	
	8–20	35 (34.4)	21(40)	14 (28)	
	>21	51 (50)	21(40)	30 (60)	
ECMO days with 38-day cut-off	<38	69 (67.6)	38 (73)	31 (62)	0.291
	≥38	33 (32.3)	14 (27)	19 (38)	
Rate of transfused PRBC units/patient/day during ECMO support	≤0.5	38 (37.3)	20 (38.4)	18 (36)	0.44
	>0.5 and <0.85	31 (30.4)	18 (34.6)	13 (26)	
	≥0.85	33 (32.4)	14 (26.9)	19 (38)	
Total amount of transfused PRBC units/patient during ECMO support	<5	21 (20.5)	12 (23)	9 (18)	0.204
	5–15	35 (34.3)	21(40)	1 (28)	
	≥16	46 (45.2)	19 (37)	27 (54)	
Total amount of transfused FFP/cryo units/patient during ECMO support	None	41 (40.3)	25 (48)	16 (32)	0.249
	<11	28 (27.4)	12 (23)	16 (32)	
	≥11	33 (32.3)	15 (29)	18 (36)	
Successful weaning from ECMO	Yes	75 (73.6)	44 (85)	31 (62)	0.013
	No	27 (26.4)	8 (15)	19 (38)	
ICU survival	Yes	62 (61)	40 (77)	22 (44)	0.001
	No	40 (39)	12 (23)	28 (56)	

ECMO—extracorporeal membrane oxygenation, VV—veno-venous ECMO, VA—veno-arterial ECMO, FFP—fresh frozen plasma, Cryo—cryoprecipitate, PRBC—packed red blood cells, NDA—no data available.

**Table 2:** A comparison of age, ECMO support duration, and blood product transfusion between ICU survivors and non-survivors (Mann–Whitney non-parametric test).

Parameters	Patient ICU Outcome	Patients, Total (*n*)	Mean ± SD	Median (Range)	*p* Value
Age, years	Total	102	48.8 ± 12.3	49.0 (17.0–73.0)	
	Alive	62	46.0 ± 12.7	45.5 (17.0–73.0)	0.001
	Dead	40	53.2 ± 10.3	54.5 (20.0–69.0)	
ECMO days	Total	102	36.3 ± 41.7	20.5 (4.0–240.0)	
	Alive	62	28.2 ± 34.5	13.5 (4.0–191.0)	0.005
	Dead	40	48.9 ± 48.6	38.5 (4.0–240.0)	
Rate of transfused PRBC units/patient/day during ECMO support	Total	102	0.76 ± 0.56	0.61 (0–2.82)	
	Alive	62	0.64 ± 0.49	0.53 (0–2.6)	0.005
	Dead	40	0.95 ± 0.62	0.86 (0.14–2.81)	
Total amount of transfused PRBC units/patient during ECMO support	Total	102	23.3 ± 28.1	12.5 (0.0–197.0)	
	Alive	62	15.4 ± 18.8	8.0 (0.0–98.0)	0.001
	Dead	40	35.5 ± 35.3	29.5 (2.0–197.0)	
Total amount of transfused FFP units/patient during ECMO support	Total	102	5.4 ± 10.0	0 (0–60)	
	Alive	62	4.7 ± 11.5	1 (0–60)	0.001
	Dead	40	6.3 ± 7.0	4 (0–28)	

ECMO—extracorporeal membrane oxygenation, PRBC—total number of packed red blood cell units, FFP—total number of fresh frozen plasma units.

**Table 3:** Bivariate analysis of factors associated with ICU mortality.

		Patients, Total (*n*)	Dead (*n)*	Mortality Rate (%)	*p* Value	OR	95% CI
Number of patients		102	40	39			
Gender	M	78	34	44		1.00	
	F	24	6	25	0.108	0.43	0.2–1.2
Age group, years	<50	52	12	23	0.004	1.00	0.0
	50–59	30	17	57	0.003	4.36	1.7–11.5
	≥60	20	11	55	0.012	4.07	1.4–12.1
Age cutoff of 50 years	<50	52	12	23		1.00	
	≥50	50	28	56	0.000	4.24	1.8–10
Type of ECMO	VV	91	33	36		1	
	VA	11	7	64	0.090	3.08	0.8-11.3
Number of ECMO days (ROC)	<38	69	19	28		1.00	
	≥38	33	21	64	0.000	4.61	1.9–11.2
Rate of transfused PRBC units/patient/day during ECMO support	≤0.5	38	9	24		1	
	>0.5 and <0.85	31	11	36	0.285	1.77	0.62–5.06
	≥0.85	33	20	61	0.002	4.96	1.78–13.79
Total amount of transfused PRBC units/patient during ECMO support	<5	21	3	14	0.000	1.00	
	5–15	35	9	26	0.319	2.08	0.5–8.8
	≥16	46	28	61	0.001	9.33	2.4–36.3
Combined effect							
Age < 50 and ECMO days	<38	38	6	16		1	
	≥38	14	6	43	0.048	4.00	1.0–15.8
Age ≥ 50 and ECMO days	<38	31	13	42		1.00	
	≥38	19	15	79	0.014	5.19	1.4–19.3
ECMO days < 38 and PRBC units/patient/day	≤0.5	26	4	15		1	
	>0.5 and <0.85	19	3	16	0.970	1.03	0.20–5.26
	≥0.85	24	12	50	0.012	5.5	1.45–20.85
ECMO days ≥ 38 and PRBC units/patient/day	≤0.5	12	5	42		1	
	>0.5 and <0.85	12	8	67	0.224	2.8	0.53–14.74
	≥0.85	9	8	89	0.046	11.2	1.04–120
Rate of transfused PRBC units/patient/day during ECMO and ECMO days	<0.85 and <38d	45	7	16		1	
	≥0.85 and <38d	24	12	50	0.004	5.43	1.74–16.9
	≤0.5 and ≥38d	12	5	42	0.058	3.88	0.95–15.76
	>0.5 and ≥38d	21	16	76	0.000	17.37	4.79–62.96

PRBC—Packed Red blood cells; OR—odds ratio.

**Table 4:** Multivariate analysis of factors associated with ICU mortality *.

Parameters	Number of Patients	*p* Value	Adjusted OR (95% CI)
Age group (years)	102		
<50	52		1.00
≥50	50	0.0003	4.47 (1.68–11.91)
Mean amount of transfused PRBC units/patient/day and ECMO days	102		
<0.85 and <38 ECMO days	45		1.00
≥0.85 and <38 ECMO days	24	0.004	5.84 (1.74–19.54)
≤0.5 and >38 ECMO days	12	0.069	3.97 (0.9–17.56)
>0.5 and >38 ECMO days	21	0.0000	17.9 (4.57–70.26)

ROC * 0.809 (0.725–0.894) PRBC—packed red blood cell units OR—odds ratio.

**Table 5:** Causes of death in the ICU.

On-ECMO	Patients, *n* = 27/102 (%)
Cardiac	3 (11.1)
Bleeding	2 (7.4)
Neurological	2 (7.4)
Mechanical complications *	2 (7.4)
Sepsis	16 (59.2)
Other	2 (7.4)
**Post-Weaning**	***n* = 13/75 (%)**
Multi-organ failure	2 (15.4)
Brain death	3 (23.1)
Respiratory failure	2 (15.4)
Sepsis	6 (46.1)

* Mechanical complications: oxygenator/tube clot, cannula dislodgement.

## Data Availability

The data that support the findings of this study are available from the corresponding author upon reasonable request.
